# Multiple Sclerosis, Viruses, and New Vaccines

**DOI:** 10.3390/neurolint13040068

**Published:** 2021-12-13

**Authors:** Peter A. C. Maple

**Affiliations:** 1 Nottingham Centre for Multiple Sclerosis and Neuroinflammation, Department of Neurology, Queen’s Medical Centre, Nottingham University Hospitals NHS Trust, Nottingham NG7 2UH, UK; eastyorksmicrobiol@gmail.com; 2 Division of Clinical Neuroscience, Queen’s Medical Centre, University of Nottingham, Nottingham NG7 2UH, UK

Multiple sclerosis (MS) is the most common inflammatory neurological disease in young adults, with an estimated prevalence of approximately 2.2 million cases worldwide [1]. Effective disease modifying treatments are increasingly available; however, a cure remains elusive [2]. The cause of MS remains to be identified, although it is evident that genetic and environmental factors play key roles [3]. There is considerable evidence [4,5] of a significant association of Epstein–Barr virus (EBV) infection with MS and, in particular, a history of infectious mononucleosis [6,7]. EBV is a human herpesvirus, which infects many children early in life [8], with few, if any, overt clinical signs or symptoms. The virus is not cleared following primary infection and a lifelong interplay is established with the host’s immune system. EBV, like all human herpesviruses, can establish a latent state with the host [9]. For those in whom EBV infection occurs later in childhood or adulthood, there is a greater risk of symptomatic infection—infectious mononucleosis. Infectious mononucleosis is characterized by lymphocytosis, pharyngitis, lymphadenopathy, and fatigue [10]. In certain circumstances, for example, when the immune control of EBV is disrupted by induced immune suppression, a very severe disease—post transplant lymphoproliferative disorder [11]—can result. EBV is also an oncogenic virus [12] and is associated with several malignancies, particularly Burkitt’s lymphoma, nasopharyngeal carcinoma, and gastric cancer [13]. For these reasons, the development of EBV vaccines is under active consideration and the last year has seen several excellent reviews published on this topic [14,15,16]. There is an increasing likelihood that EBV vaccines may become available, raising the possibility of prophylactic vaccination to prevent MS.

There is emerging evidence that infection by another human herpesvirus—cytomegalovirus (CMV)—may be negatively associated with the development of MS. Several studies [17,18,19] have reported significantly lower seroprevalences of CMV IgG in people with MS compared to non-MS controls. By adulthood, many people have been infected by CMV with no apparent health consequences and infection is lifelong due to the capacity of the virus to establish latency. Although CMV can infect several cell types, latency is established in haematopoietic progenitor cells and monocytes of the myeloid lineage [20]. In cases where the immune response is compromised, either by induced immunosuppression for transplantation or infection (e.g., HIV), CMV infection can be a major clinical problem. Furthermore, congenital CMV infection can lead to abnormal foetal development and is a leading cause of sensorineural hearing loss. For these reasons, CMV vaccines are under active development [21,22] raising the possibility of a potential application for the prevention of MS.

The responses of people with multiple sclerosis to routine vaccinations have been a topic of considerable interest given the use of disease-modifying therapies (e.g., anti-CD20) that directly impact the host’s immune response [23,24]. Particularly relevant is the need to vaccinate against COVID-19, which has resulted in the publication of several guidelines [25,26,27]. Severe acute respiratory syndrome coronavirus-2 (SARS CoV-2) is the causative agent of COVID-19 and infections range from asymptomatic to life threatening. Another facet of SARS CoV-2 infection is the development of neurological manifestations, both in the short term and over longer time periods [28]. It has been suggested that EBV reactivation may be a key contributor to the development of the longer-term neurological sequelae [29]. Vaccination has proven to be the most effective means of preventing many infectious diseases, including COVID-19, and in the context of multiple sclerosis prophylactic and therapeutic vaccination [30] may yield several future benefits.
